# The pattern of symptoms in patients receiving home based care in Bangwe, Malawi : a descriptive study

**DOI:** 10.1186/1472-684X-5-1

**Published:** 2006-02-10

**Authors:** Cameron Bowie, Linda Kalilane, Paul Cleary, Claire Bowie

**Affiliations:** 1 Department of Community Health, College of Medicine, University of Malawi, Blantyre, Malawi; 2 Johns Hopkins Research Project, College of Medicine, Blantyre, Malawi; 3 Department of Public Health, University of Liverpool, UK; 4 Salvation Army Bangwe Project, Blantyre, Malawi

## Abstract

**Background:**

Home based care of HIV/AIDS patients is a health need recommended but not often available in Africa. Population based assessment helps to identify unmet health needs to plan services. Careful assessment and follow up of patients receiving home based care in a defined population of Bangwe, Malawi provides details of the frequency and severity of common symptoms.

**Methods:**

Mortality and the incidence, duration and severity of common symptoms of patients in a defined population receiving home based care were measured over an eighteen month period.

**Results:**

358 patients, of whom 199 died, were studied. A third of patients died within 4 months of being first seen. About half the patients were unable to care for themselves on first assessment. Half were malnourished with a Body Mass Index (BMI) < 18.5 kg/m^2^. Most patients had a mixture of symptoms at presentation. These symptoms responded to treatment usually within a fortnight. However a small proportion (5%) of patients suffered repeated episodes often as many as 6–9 times a year. Incidence rates are estimated.

**Conclusion:**

Symptoms which are alleviated by simple treatments are common. The patients in whom symptoms recur need a responsive home based care service. Population based estimates of incidence and duration of disease and the visit work load allow an assessment of home based care needs in an urban setting in Africa.

## Background

Home based care of people living with AIDS is an established component of the continuum of care and support advocated by WHO and UNAIDS [1] and planned by many African countries including Malawi [2]. The pattern of health problems as the disease progresses will vary from one population to another [3]. An understanding of the incidence and duration of common illnesses helps to assess the needs of communities for home based care [4].

The Bangwe project is a joint home based care (HBC) project run by the Salvation Army and the Department of Community Health, College of Medicine, University of Malawi. While providing a standard HBC service to the people of Bangwe, a township of 40,000 people adjacent to the town of Limbe near Blantyre, the opportunity is taken to collect data on the health problems of patients, their response to treatment and their nutritional status. Antiretroviral drugs were not being used during the study period, which was a time before antiretroviral therapy became available free of charge in Malawi.

The project has been collecting patient data since January 2003. The project has been assessing the value of the national standard treatments for home based care as promulgated by the National AIDS Commission (NAC), and simultaneously assessing the effect of supplementary feeding for the World Food Programme (WFP) [5].

This report summarises the results of 18 months work assessing clinical care and patient careers. It is an example of operations research in "standard" health care on offer in Malawi. The detailed records kept and transferred to a database allow detailed analysis of the natural history of patients enrolled in a home based care scheme and the responses to treatment of basic palliative care and simple treatments of opportunistic infections common in people with terminal AIDS. The report does not encompass other equally important aspects of support and counselling provided by the team and community volunteers, which have been described in the Southern African context [6,7].

The frequency and severity of symptoms reported by people with HIV/AIDS has been published in various settings. A study of HIV patients receiving home based care in New York in 1991 before the advent of Highly Active Antiretroviral Therapy (HAART) found the frequently observed signs and symptoms included dyspnoea, weakness, fatigue/lethargy, pain, ataxia, cough, skin lesions, and memory deficit [8]. Holzemer reports the results of a US national survey of people living with HIV/AIDS after the introduction of HAART when the commonest symptoms were anxiety and fear (17.3%), diarrhoea (16%), peripheral neuropathy (11.6%), nausea and vomiting (9.7%), depression (8.1%) and fatigue (7%) [9]. He reports that greater frequency and intensity of symptoms leads to a lower quality of life.

The study population has been well demarcated which allows estimates of disease frequencies, severity levels of symptoms, rates of recurrences and unmet need rates and the duration of episodes. These can be used to extrapolate for planning purposes.

## Methods

### The project

The study area is comprised of four villages in Bangwe with an estimated population of 23,044 at the last census taken in 1998, and now thought to have grown to 26,500 based on a census carried out in part of the area in 2003 which found a population growth of 15% in the five years since the national census. Antenatal HIV seroprevalence was 28% in Limbe in 2003.

The home based care project forms part of a Salvation Army HIV/AIDS Community project which encompasses community action concerning prevention, orphan care and home based care. For the home based care, community volunteers are recruited and trained in basic home based care as devised by the NAC. There are usually two home based care volunteers for each village. They identify people who may need home based care, and if requested, refer the patient to the home based care team. They accompany the member of the team to see the patient, offer to undertake support tasks if the patient has no carer, and do follow up visits to assess progress and future needs.

The team, two nurses and two home based care assistants, supported by the Department of Community Health of the College of Medicine, provides the clinical care in line with national guidelines. An initial assessment form [Additional file 1] listing the common symptoms and signs is completed by the nurse at the first visit. The form includes, for instance, detailed information about site and severity of pain using a visual analogue scale, which together with the type of pain is used by the nurse to assess the care needs of the patient. When pain is severe at the first visit, the patient is given enough non opioid analgesia until the next planned visit. The patients who do not respond to non opioid analgesia are given morphine.

Patients are followed up usually weekly or fortnightly after initial assessment depending on patient's needs and a follow up form is completed. The site, type and severity of pain are monitored at every visit, though nurse records are not entered into the data file (due to resource constraints).

Each nurse carries a standard kit containing dressings and drugs and these are dispensed free of charge. Where neuropathic pain is present a combination of Brufen and Amitriptyline is given. Where there is possible Isoniazid induced peripheral neuropathy a therapeutic dose of Pyridoxine is given for 2–3 weeks followed by a low continuation dose.

Two particular drugs (loperamide and amytriptyline) were introduced into the kit and made available in the course of the study. The opportunity has been taken to compare the responses to these two drugs to the standard treatment used before their introduction (analgesics for neuropathic pain and codeine phosphate for diarrhoea).

Data have been collected since the project started in January 2003 from information collected at the time of initial assessment and at follow up visits of individual patients. Inclusion criteria are that patients are aged between 15 and 50 years with chronic disease of more than a month, and in need of home based care. Data were entered once onto an excel spreadsheet, checked and SPSS used for routine statistical analysis.

Clinical staging of HIV/AIDS is based on current WHO guidelines [10]. The severity of pain at different sites was assessed using a visual analogue scale of 1 to 10, with 1 being mild and 10 severe. A pain score of 1 to 4 was classified as mild, 5 to 7 as moderate and 8 to 10 as severe.

This study was an audit of standard care and as such did not require ethics research committee approval.

## Results

### Demographic characteristics

**Figure 1:**
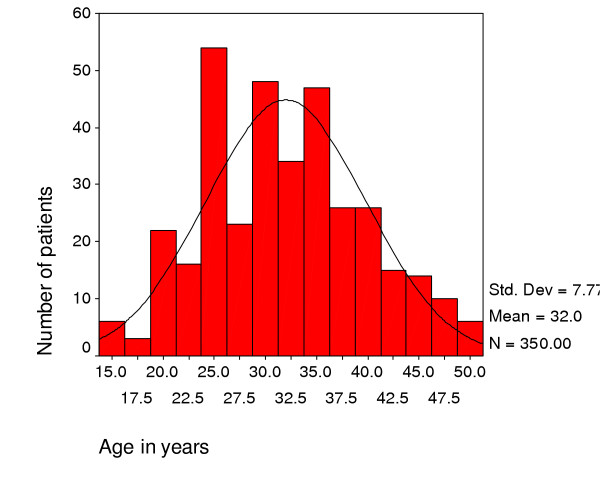
Age distribution of home based care patients, Bangwe Malawi.

358 patients were enrolled of which 59% were women. The age distribution of patients follows one well recognised in AIDS patients with a mean age of 32 years (Figure 1). Females were younger (mean age of 30.9 years) than males (mean age of 33.4 years).

A slight majority of patients (52%) are married, a third (35%) are either divorced or widowed and 13% single, which may compromise household caring capacity. The average number of people in the households was 5.4 of who 1.2 were children under 5 years of age.

**Figure 2:**
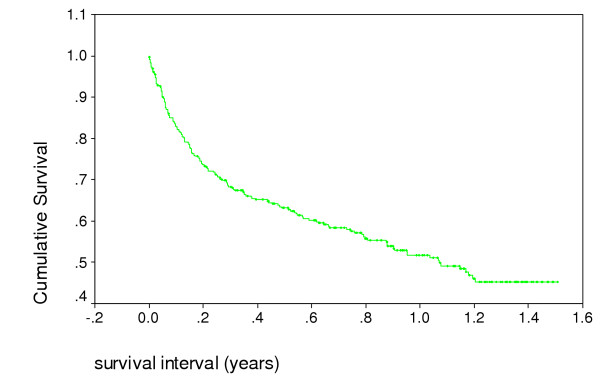
Survival of home based care patients, Bangwe Malawi – Kaplan Meier analysis.

Many patients presented in advanced stage of disease. A third of patients died within 4 months of being first seen. Half survive one year (Figure 2). In the 18 months of the study 199 of the 358 patients have died (56%).

A household census of part of the area carried out in 2003 found 7.8 per 1000 aged between 13–49 years were chronically sick (for more than a month). This equates to 205 chronically sick of this age group living in the study area. At the time of the census 142 patients had been enrolled into the HBC service of which 97 were still alive. It appears that about half the chronic sick in the study area were enrolled at the time.

### HIV status

Voluntary counselling and testing (VCT) is becoming more popular. Of the home based patients seen at first assessment a quarter (25%) had been tested. Just over half (54%) of those tested reported a positive test. This may be an underestimate due to the test being carried out before infection, or due to incorrect result recall perhaps due to stigma attached to HIV status? Clinical assessment suggests that 95% of patients have AIDS related disease. A majority who had not had a test said they wanted to have one. VCT became available at the local health centre towards the end of the study period.

### Treatment prior to assessment

TB had been treated in 31% of patients who had been diagnosed on average 99 days before being seen by the home based care team. Many (41% of) patients had tried traditional medicine. Over half had been in hospital in the previous 5 years. For these the average number of admissions was 2.3 in the last five years and the estimated length of stay was 23 days.

The estimated cost of drugs bought in the previous four weeks was 452 kwacha ($5). These were mainly obtained either from hospital (40%) or from street vendors (39%).

### Clinical staging and nutritional status

Most patients have HIV/AIDS and less than 5% have other chronic conditions such as paraplegia. The majority of patients presented in an advanced stage of disease, with 70% in WHO stage 4, 25% in stage 3 and 5% in stage 2. None presented with WHO stage 1 disease.

**Figure 3:**
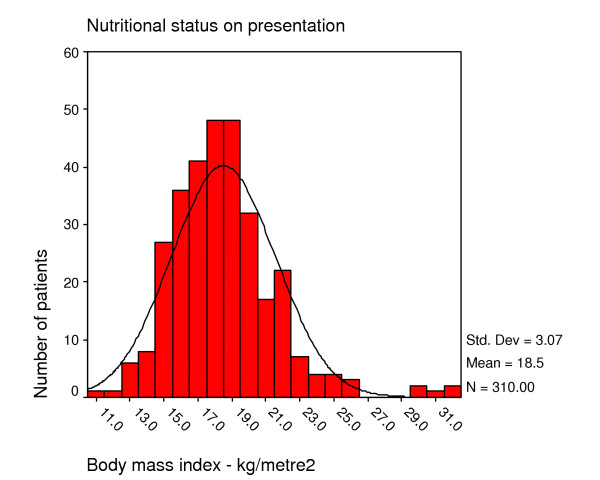
Nutritional status of home based care patients on first presentation, Bangwe Malawi.

Nearly all patients (94%) thought they had lost weight. Of those who could remember their healthy weight, the average weight loss was 12 kg. The mean BMI at presentation was 18.5 kg/m2 (SD 3.1). Half the patients were malnourished with a body mass index (BMI) of less than 18.5 kg/m2 on enrolment. A quarter was severely malnourished below 16 kg/m2 (Figure 3).

### Activities of daily living

Just under half (44%) of the patients at initial assessment were able to care for themselves. Indeed, only 15% said they were able to live normally. Many had multiple needs for caring support (Table 1). A third needed help with washing or walking, a quarter with the toilet, 17% with dressing, and 9% with eating. In the previous seven days three quarters (76%) had spent more than half of each day in bed.

**Table 1:** Activities of daily living of patients on first enrolment in home based care service

**Activity of daily living question**	**Number saying "yes"**	**Percentage**
Able to continue normal activity?	54	15%
Able to leave house?	259	72%
Needs help for normal living?	160	44%
Needs help with washing?	124	34%
Needs help with dressing?	61	17%
Needs help with eating?	31	9%
Needs help with walking?	127	35%
Needs help with toilet?	100	28%

### Symptoms

Multiple symptoms were often present on first assessment. The common symptoms were headache, fever, chest pain, shortness of breath and cough. Details of each symptom with, for selected symptoms, the duration of symptom at first assessment, the duration of the first episode and the number of repeat episodes per year are found in Table 2. All symptoms have skewed distributions for their duration and frequency. The 90^th ^or 95^th ^percentile values show the long durations and high frequencies suffered by a few.

**Table 2:** The number, frequency duration, severity of presenting symptoms of patients at initial assessment by the home based care service

**Symptom on first presentation**	**Number and Percentage of patients with symptom^a^**		**Duration of symptoms prior to presentation in days^a^**			**Persistence of symptom after treatment dispensed at time of initial assessment in days^b^**			**Frequency of recurring symptom per year^b^**		
	number	%	median	75 percentile	90 percentile	median	75 percentile	95 percentile	median	75 percentile	95 percentile

Headache	267	74				14	29	189	1.9	3.2	7.7
Fever	253	70	4	14	52	14	35	117	1.7	4.0	9.4
Chest pain	245	68				14	42	167	2.2	4.7	8.9
Shortness of breath	241	67									
Cough	239	66	4	15	36	19	45	150	2.6	4.1	9.1
SOB walking	221	61									
Stomach pains	215	60									
Poor appetite	205	57				12	21	92	0.9	3.4	7.9
Lower limb pain	163	45				28	68	199	1.6	3.2	7.1
Nausea or vomiting	143	40									
Diarrhoea	140	39	3	12	52	7	14	70	0.0	3.4	9.4
Problem swallowing	121	34									
Skin problems	122	34									
SOB doing nothing	107	30									
Thrush	89	29				8	27	195			
Previous shingles	101	28									
Other pain	97	27									
Itchy rash	93	26				14	50	298	0.0	1.3	4.5
Genital ulcers	46	13									
Urethral discharge	40	11									
Mouth ulcers	37	10									

Note ^a ^calculated from initial assessment data

^b ^calculated using follow up data

Most patients (95%) were in pain of some sort at first assessment with 84% experiencing moderate or severe pain (Table 3). In the majority of cases this became well controlled with non opioid analgesia. Symptom control and support of other HIV/AIDS related problems usually contributed to the patient's comfort. Where pain control was not adequate with this combination, liquid Morphine was used.

**Table 3:** Presence of pain at any site at initial assessment by the home based care service

**Presence and severity of pain at any site**	**Number (n = 360)**	**Percentage**
No pain	18	5
Mild pain	41	11
Moderate pain	117	33
Severe pain	184	51

Data on the duration of symptom prior to assessment were only collected for fever, cough and diarrhoea. While the usual episode had only been present for a few days, a few (10%) had suffered the symptoms for over a month.

While treatment was effective in alleviating symptoms usually within a fortnight, a quarter took 3–6 weeks and a small number (5%) of patients' symptoms were unresponsive to the treatment on offer for over 6 months. Itchy rash, headache and chest pain were particularly obdurate.

While the majority of patients had recurrent episodes of their presenting symptoms twice in a year, a small number (5%) had repeat episodes about every 6 weeks.

### Selected specific treatments

#### Amitriptyline

Neuropathic pain associated with peripheral neuropathy is a particularly persistent symptom, usually of the lower limb. Amitriptyline was introduced in September 2003 and is given along with analgesics for this symptom. The duration of initial episode of this symptom was less once amitriptyline was used in combination with a analgesic, though not statistically significant, reducing from a mean duration of 78 to 59 days, and a reduction in recurrences from 2.7 to 1.9 episodes per year.

#### Anti-diarrhoeal disease

Loperamide is cheaper than codeine phosphate in Malawi. There is a difference in response to the alternative drugs in patients which is not statistically significant. Codeine phosphate is associated with shorter duration of diarrhoea on first assessment and with fewer episodes per year (Table 4).

**Table 4:** Response times to treatment and recurrences of diarrhoea

	Number	Mean	95% confidence interval for mean	
			Lower	Upper
**Duration of first episode in days**				

Both codeine phosphate and loperamide	15	34.3	-6.8	75.5
Codeine phosphate	29	20.1	11.5	28.8
Loperamide	5	26.1	13.1	39.1

**Episodes per year**				

Both codeine phosphate and loperamide	45	4.8	1.7	8.0
Codeine phosphate	63	2.8	1.3	4.3
Loperamide	16	6.5	-0.7	13.7

### Home based care team workload

Detailed workload statistics are available for the 18 month period (Table 5). For the 358 patients initially assessed, a quarter (26%) was not followed up, either because the patient died, was admitted to hospital, moved away or did not require such a visit. The average number of follow up visits is 6.1, the median is four and the mode is one. For workload planning purposes, for a total population (all ages) of 1000 persons, there will be 9 new patients and 37 follow up visits a year (Table 6).

**Table 5:** patient visits in 18 months

Type of visit	visit number	Frequency	Percent
Initial visit	0	358	100%
Follow up visit	1	264	74%
	2	209	58%
	3	168	47%
	4	139	39%
	5	112	31%
	6	96	27%
	7	81	23%
	8	72	20%
	9	58	16%
	10	48	13%
	11	39	11%
	12	34	9%
	13	28	8%
	14	24	7%
	15	20	6%
	16	18	5%
	17	13	4%
	18	11	3%
	19	8	2%
	20	6	2%
	20+	41	11%
Total follow up		1489	
All visit		1847	

**Table 6:** Workload of home based care

	18 month study period	12 months equivalent number	Study population	Visit rate per 1000 total population
New patients	358	239	26500	9
Follow up visits	1489	993	26500	37
Total visits	1847	1,231	26500	46

## Discussion

The morbidity and mortality of this group of patients is high. The vast majority were not receiving antiretroviral therapy. The results provide a measure of the morbidity and mortality of AIDS without such therapy. Needs are high with multiple symptoms presenting on enrolment and a number of symptoms recurring. Although some patients die soon after being seen for the first time, others survive with need for long term clinical care and palliative therapy. The range and frequency of symptoms are not unexpected, and similar to pre-HAART care elsewhere. For instance 84% of patients reported moderate or severe pain on first assessment. This can be compared to the experience in Uganda where 80% of cancer patients reported pain [11]. The prevalence of substantial pain is higher than found in other African countries such as the estimate (25%) provided for HIV/AIDS patients in Uganda. Although most respond to non-opioids or an NSAID, some require an opioid (codeine phosphate). However codeine phosphate is expensive and often unobtainable and morphine becomes the drug of choice. At any one time there are usually one or two patients on morphine. Unfortunately, the use of morphine has its limitations in urban settings such as Bangwe. The administration of oral morphine can be difficult to manage and monitor. Households seldom have a clock or watch. The concept of regular, 4 hourly dosage is difficult to understand and remember. Guardians are suspicious of liquid morphine and a syringe and often stop giving it. HBC volunteers are unable or unwilling to visit more than once a day if carers are unable or unwilling to give the drug. Oral morphine has a short shelf life. A morphine modified release preparation (MST), although currently expensive, would be a useful alternative in certain situations if a cheap formulation was available.

Simple treatment of opportunistic infections seems to be effective for most but not all symptoms. The majority of symptoms continue for about a fortnight so it is appropriate to prescribe adequate quantities of drugs to last this time. Weekly visits are required for patients requiring palliative care or those with severe infections and the visits continue until symptoms are controlled. Repeat episodes after treatment tend to last shorter than the duration of the symptom before treatment. Recurrences are not usual but do occur in a few patients. The role of the community volunteer in identifying these patients is therefore important.

Issues of nutrition are considered in a parallel paper [5]. Moderate and severe malnutrition are common but food supplementation as offered by the WFP does not seem to help survival, nutritional status or symptoms. Clearly, like terminally ill patients in Uganda [12] and South Africa [6], other needs are prevalent with food being only one. Income relief is probably more appropriate than just food in urban settings, and this needs to be studied.

The census undertaken in part of the study population area indicates that about half those with chronic illness will request help from the home based care team. Some will not seek help for a variety of reasons. The better the community volunteers, the better the coverage. For planning purposes it is reasonable to assume a similar proportion will not need or seek such care. The rates which apply in Bangwe can be used with confidence for planning home based care services in urban areas in Malawi and countries with similar HIV prevalence.

## Conclusion

The need for home based care is confirmed at a level which is not currently provided in most of Malawi. Symptoms are common, can last a considerable time in a small proportion of patients and can usually be alleviated using simple treatment. The study provides a baseline of the pattern of the disease which can be used to assess the effect of antiretroviral therapy as it becomes more widely available.

## Competing interests

The author(s) declare that they have no competing interests.

## Authors' contributions

CB conceived the survey and analysed and wrote the first draft. PC designed the survey and undertook preliminary analysis. CTB undertook and supervised the data collection. LA supervised data entry. All contributed to the final report.

## Pre-publication history

The pre-publication history for this paper can be accessed here:



## Supplementary Material

Additional File 1 the form used on initial assessment of the patient. Click here for file
