# Supplementary material for: The pattern of symptoms in patients receiving home based care in Bangwe, Malawi : a descriptive study
Source: BMC Palliat Care. 2006 Feb 10;5:1. doi: 10.1186/1472-684X-5-1 (PMC1382199; doi:10.1186/1472-684X-5-1)
Supplement: Additional File 1 — the form used on initial assessment of the patient. [file 1472-684X-5-1-S1.doc]

1) Code number:

/ /

2) Date of assessment (dd/mm/yy):

3) Initials of assessor:

4) Initials of translator:

5) Family name of subject:

6) First name:

/ /

7) Date of birth (dd/mm/yy):

8) Approximate age:

9) Sex: MALE 1

FEMALE 2

10) Marital status: NEVER MARRIED 1

CURRENTLY MARRIED 2

DIVORCED 3

WIDOWED 4

11) Religion: PROTESTANT 1

CATHOLIC 2

MUSLIM 3

OTHER 4

12) Location: MWAMADI 1

NKHUKUTENI 2

WISIKI 3

KAWINGA 4

MASALA 5

MONDIWA 6

GOMANJIRA 7

CHILAMBE 8

OTHER (please specify) 9

13) Total number of people of all ages permanently resident in household:

14) Number of children aged < 5:

15) Is the patient well enough to answer questions? YES 1

NO 2

16) Is the patient able to continue normal activities? YES 1

NO 2

YEARS

17) How long since the patient last felt completely well? MONTHS

WEEKS

18) Is the patient able to leave the house without help? YES 🡺 *QUESTION 20* 1

NO 2

YEARS

19) How long ago could the patient last leave the house without help?

MONTHS

WEEKS

20) At the moment, does the patient need

help from another person to: - WASH? YES 1

NO 2

- DRESS? YES 1

NO 2

- EAT? YES 1

NO 2

- WALK? YES 1

NO 2

- GO TO THE TOILET? YES 1

NO 2

21) How many hours of the day

is someone nearby who can help the patient?

*(Excluding community volunteers.)*

22) In the last seven days,

how much of the day has the patient spent lying down? LESS THAN HALF THE DAY 1

MORE THAN HALF THE DAY 2

ALL DAY 3

DON’T KNOW 4

23) Has the patient lost weight? YES 1

NO 2

24) Does the patient know

what their weight was when they were last fit and well? YES 1

NO 🡺 *QUESTION 26* 2

25) Weight when last fit and well:

26) Does the patient have fever at the moment? YES 1

NO 🡺 *QUESTION 28 2*

YEARS

27) How long has the patient had fever for? MONTHS

WEEKS

28) Does the patient suffer from chest pain? YES 1

NO 🡺 *QUESTION 30* 2

29) How bad is the pain without painkillers?

30) Does the patient get short of breath? YES 1

NO 🡺 *QUESTION 33* 2

31) Does the patient get short of breath when walking? YES 1

NO 2

32) Does the patient get short of breath when doing nothing? YES 1

NO 2

33) Has the patient suffered from a cough? YES 1

NO 🡺 *QUESTION 37* 2

34) How long has the patient suffered from a cough? YEARS

MONTHS

WEEKS

35) Is the cough productive of sputum? YES 1

NO 2

36) Has the patient coughed up blood? YES 1

NO 2

37) Has the patient had a poor appetite? YES 1

NO 2

38) Has the patient vomited or felt nauseated? YES 1

NO 2

39) Has the patient vomited blood? YES 1

NO 2

40) Has the patient had pain or difficulty when swallowing? YES 1

NO 2

41) Has the patient had stomach pains? YES 1

NO 🡺 *QUESTION 44* 2

42) Is the pain associated with food? YES 1

NO 2

43) How bad is the pain without painkillers?

44) Has the patient had diarrhoea? YES 1

NO 🡺 *QUESTION 47* 2

YEARS

45) How long has the patient had diarrhoea? MONTHS

WEEKS

46) When the patient has diarrhoea, roughly how many times do they open their bowels in 24 hours?

47) Has the patient been constipated? YES 1

NO 🡺 *QUESTION 49* 2

48) When the patient has constipation, roughly how many times do they open their bowels in seven days?

49) Has the patient passed blood in their stool? YES 1

NO 2

50) Has the patient had problems passing water? YES 1

NO 2

51) Has the patient passed blood in their urine? YES 1

NO 2

52) Has the patient had a headache? YES 1

NO 🡺 *QUESTION 54* 2

53) How bad is the pain without painkillers?

54) Has the patient had any other pains? YES 1

NO 🡺 *QUESTION 62* 2

*Record pain score below for each pain mentioned.*

55) NECK

56) TORSO

57) UPPER LIMBS

58) ABDOMEN

59) PELVIS

60) LOWER LIMBS

61) ALL OVER

62) Has the patient had any problems with their skin? YES 1

NO 🡺 *QUESTION 65*  2

63) Does the patient have an itchy rash? YES 1

NO 2

64) Does the patient have any bedsores? YES 1

NO 2

65) Has the patient ever had shingles? YES 1

NO 🡺 *QUESTION 67* 2

YEARS

66) How long ago did they have shingles? MONTHS

WEEKS

67) Has the patient ever had TB? YES 1

*If more than one episode, collect data on latest episode.*

NO 🡺 *QUESTION 73* 2

*(Specify if PTB or EPTB – not for analysis)*

YEARS

68) How long ago was it diagnosed? MONTHS

WEEKS

69) Did they have treatment for TB? YES 1

NO 2

70) What year was TB treatment started?

71) How many months of TB treatment did the patient receive, or, if still on treatment, how many

months of treatment have they received so far?

72) Is the patient still on TB treatment? YES 1

NO 2

73) In the last five years, has the patient ever been admitted to hospital? YES 1

NO 2

74) How many times?

75) How many days do you think they have spent in hospital over the last five years?

76) *Ask the patient what medicines they have taken in the last four weeks.* *Ask to see drug packaging to corroborate and / or complete medication history and give generic names where available.*

77) Where does the patient get these medications?

*(1=YES, 2=NO).*

HOSPITAL 1 2

HEALTH POST 1 2

PRIVATE DOCTOR 1 2

PHARMACY 1 2

STREET SELLER 1 2

78) How much has the patient spent on medications in the last month?

79) Has the patient taken any traditional medicines in the past four weeks? YES 1

NO 2

80) Has the patient drunk any alcohol in the past four weeks? YES 1

NO 2

81) *Inform the patient that they do not have to answer this question.*

Has the patient ever had an HIV test? YES 1

NO 🡺 *QUESTION 84* 2

82) What was the result? POSITIVE 1

NEGATIVE 2

83) How long ago was the patient tested? YEARS MONTHS

WEEKS

84) Does the patient wish to have an HIV test? YES 1

NO 2

85) Does the patient have genital ulceration? YES 1

NO 2

86) Does the patient have penile / vaginal discharge? YES 1

NO 2

87) *Ask this question only if the patient is female* YEARS*.*

How long is it since the patient last had a menstrual period?

MONTHS

WEEKS

88) *Ask this question only if it is more than four weeks since the patient’s last menstrual period.*

Does the patient think they may be pregnant? YES 1

NO 2

*Thank the patient for answering the questions.*

*Ask to see patient’s medical card and record any relevant information (not for analysis).*

*Examine the patient’s mouth. Is there evidence of…?*

89) MOUTH ULCERS YES 1

NO 2

90) THRUSH YES 1

NO 2

91) ORAL HAIRY LEUKOPLAKIA YES 1

NO 2

*If there are any other examination findings of note, record here and space for notes (not for analysis).*

*Record anthropomorphic measurements of patient.*

92) WEIGHT (kg)

93) HEIGHT (cm)

94) MUAC (cm)

95) *Record names and ages of other household members. Record anthropomorphic measurements of those household members available.*

| DATE (dd/mm /yy) | LAST NAME | FIRST NAME | DOB (dd/mm/yy) | AGE (years) | SEX | WEIGHT (kg) | HEIGHT (cm) | MUAC (cm) |
| --- | --- | --- | --- | --- | --- | --- | --- | --- |
|  |  |  |  |  |  |  |  |  |
|  |  |  |  |  |  |  |  |  |
|  |  |  |  |  |  |  |  |  |
|  |  |  |  |  |  |  |  |  |
|  |  |  |  |  |  |  |  |  |
|  |  |  |  |  |  |  |  |  |
|  |  |  |  |  |  |  |  |  |
|  |  |  |  |  |  |  |  |  |
|  |  |  |  |  |  |  |  |  |
|  |  |  |  |  |  |  |  |  |
|  |  |  |  |  |  |  |  |  |
|  |  |  |  |  |  |  |  |  |
|  |  |  |  |  |  |  |  |  |
|  |  |  |  |  |  |  |  |  |

*Continue on separate sheet if necessary.*

96) *Record drugs prescribed on this visit.*

97) *Record if wound care given.* YES 1

NO 2

98) *Record if patient referred for VCT.* YES 1

NO 2

99) *Record if patient referred to hospital.* YES 1

NO 2

100) *Record if sputum samples sent for AFB.* YES 1

NO 2

101) *Record any other action taken and date of next visit*
